# Multilevel Societies in Primates and Other Mammals: Introduction to the Special Issue

**DOI:** 10.1007/s10764-012-9614-3

**Published:** 2012-06-08

**Authors:** Cyril C. Grueter, Ikki Matsuda, Peng Zhang, Dietmar Zinner

**Affiliations:** 1 School of Anatomy, Physiology and Human Biology, The University of Western Australia, Crawley, WA 6009 Australia; 2 Anthropological Institute and Museum, University of Zürich-Irchel, 8057 Zürich, Switzerland; 3 Primate Research Institute, Kyoto University, Inuyama, 484-8506 Japan; 4 Anthropology Department, Sun Yat-sen University, Guangzhou, 510275 China; 5 Cognitive Ethology Laboratory, German Primate Center, 37077 Göttingen, Germany; 6 Courant Research Centre “Evolution of Social Behavior”, University of Göttingen, 37077 Göttingen, Germany

Murdoch (1981) remarked that nowhere on Earth do people live regularly in isolated families. The habitual formation of superfamily level groupings is one of the unmistakable universals of human sociality. Males and females within these higher level groupings are connected via kinship and affinity ties, and affiliative and cooperative bonds reach far beyond the nuclear family unit (Rodseth *et al*. 1991; Wiessner 1977). Although interunit encounters are usually circumvented or characterized by animosity because of mating or resource competition in many nonhuman primates (Cheney 1987; Fashing 2001), some primates such as hamadryas baboons (*Papio hamadryas*), geladas (*Theropithecus gelada*), snub-nosed monkeys (*Rhinopithecus* spp.), and proboscis monkeys (*Nasalis larvatus*) (for an exhaustive list, see Grueter *et al*. 2012) exhibit a social arrangement in which regular or constant proximity as well as coordinated activity among subunits is the norm. This type of social organization has been termed multilevel, nested, or modular. The phenomenon of social modularity is not restricted to the primate order. Similarly structured societies can be observed in other mammals, most notably African elephants (*Loxodonta africana*: de Silva and Wittemyer 2012; Moss and Poole 1983; Wittemyer *et al*. 2005), Asian elephants (*Elephas maximus*: de Silva and Wittemyer 2012), plains zebras (*Equus burchelli*: Rubenstein and Hack 2004), khulans (*Equus hemionus*: Feh *et al*. 2001), prairie dogs (*Cynomys ludovicianus*: Hoogland 1995), sperm whales (*Physeter macrocephalus*: Whitehead *et al*. 1991, 2012), and killer whales (*Orcinus orca*: Baird 2000).

Stammbach (1987) was the first to compare the social organization and social interactions of baboon species exhibiting multilevel societies. Later, Grueter and Zinner (2004) elaborated on this earlier review by contrasting modularity of papionins with that of snub-nosed monkeys —a hitherto poorly known primate radiation— and synthesizing information on various aspects of their respective social systems. With the present special issue, we have brought together experts in the field of multilevel sociality for the first time with the aim of tackling the issue of the evolution of multilevel societies from various angles and adopting a broad-scale approach by including papers on species other than primates as well as contributions by sociocultural anthropologists. Here we aim to introduce the phenomenon of multilevel societies and give a synopsis of the range of topics covered by the contributions to this theme issue.

Multilevel societies are typically characterized by discrete social stratification with at least one stable core unit. de Silva and Wittemyer (2012), however, call for a relaxed definition of multilevel societies to encompass not only species showing such nestedness, but also systems in which an individual associates with more than one set of companions and societies in which levels are less clearly delineated and transition more gradually. It has become apparent that in nonprimate multilevel societies, such as those of cetaceans and proboscideans, boundaries between tiers are not as sharp as in primates, with the possible exception of uakaris (Bowler *et al*. 2012). In primates, the most common module at the base of a multilevel society is a one-male–multifemale unit (OMU) or “harem,” with all-male units often being in the neighborhood (Grueter and Zinner 2004). In other mammals, the core unit can also be closely associated breeding females, as seen in elephants (Wittemyer *et al*. 2005) or sperm whales (Whitehead 2003; Whitehead *et al*. 1991, 2012). Although a classic multilevel society is typically composed of two to four hierarchically inclusive social tiers (Kawai *et al*. 1983; Ren *et al*. 2000), in some species, such as elephants, the picture is more complicated, with up to six identified tiers (Wittemyer *et al*. 2005). Hamadryas baboons —at least the Filoha population— also seem to have an impressive five different levels (Schreier and Swedell 2012). More thorough research may well reveal previously hidden levels of complexity in the architecture of multilevel social systems.

Multilevel societies are superficially the most complex social systems found among primates or mammals in general. To understand how these societies work, we need to investigate how social interactions and relationships are patterned both within and between social elements, and analyze the kinship structure of the population, i.e., the degree of genetic relatedness among individuals within and between units, which is contingent on dispersal patterns. The diversity in the cohesiveness and nature of intrafamily social bonds —with some of the core units being female-bonded and others cross-sex bonded (Grueter *et al*. 2012; Matsuda *et al*. 2012; Zhang *et al*. 2012)— adds to the intricacy of the system.

A prerequisite for understanding multilevel sociality is a formal analysis of the composition of the group. Social levels need to be clearly defined and boundaries delineated (Kawai *et al*. 1983; Snyder-Mackler *et al*. 2012). State-of-the-art tools such as social network analysis and hierarchical cluster analysis facilitate analyses of the structural properties of multilevel organizations (Matsuda *et al*. 2012; Snyder-Mackler *et al*. 2012; Wittemyer *et al*. 2005; Yeager 1990; Zhang *et al*. 2012; see also Schweizer 1997). However, even identifying a multilevel system as such has sometimes proven difficult, as it requires good observation conditions (open terrain and terrestrial behavior) at the level of the individual or at least the unit (Bowler *et al*. 2012). Given the sheer size of many multilevel primate groups (often with up to several hundred members), achieving individual identification is a challenge under wild and unprovisioned conditions. Indeed, some multilevel societies were formerly considered to be macaque-like multimale–multifemale groups (Kawabe and Mano 1972; Li *et al*. 1982). Data on the spatiotemporal distribution of individuals can help to detect the boundaries of the core units (Grueter *et al*. in press). Recognizing the boundaries of intermediate levels (if present) is much more difficult, especially in gelada and Guinea baboon groups which fission and fuse throughout the day in an almost seamless manner (Dunbar 1993; Patzelt *et al*. 2011; Snyder-Mackler *et al*. 2012).

Multilevel societies might easily be confounded with aggregations, i.e., situations in which animal units are attracted by extrinsic stimuli such as clumped food or roosts. Although a number of elephant seal harems sharing a beach or western gorillas units coming into a bai to forage might on the surface resemble a multilevel society, these are in fact nonmutualistic assemblages in which individuals do not actively maintain proximity as a means of obtaining benefits from each other’s presence (*cf*. Connor 2000). Similarly, hamadryas baboon gravitate to scarce sleeping sites in their habitat, but the ensuing troop level does not qualify to be considered a “social” unit in the strict sense. Similarly, when two multimale–multifemale groups of olive baboons share a rocky cliff as a sleeping site in areas without suitable trees, we would not classify this as a multilayered organization. It is also worth noting that multilevel societies are not the same as fission–fusion systems. Fission–fusion is common in many multilevel societies, e.g., sperm whales (Christal *et al*. 1998), hamadryas baboons (Kummer and Abegglen 1978), or snub-nosed monkeys (Ren *et al*. 2012), but fission–fusion dynamics can characterize both species living in multilevel societies and multimale–multifemale groups (Grueter *et al*. 2012).

Although a single social grouping serves a variety of functions in nonmodular species (predator and food defense, social bonds and cooperation, mating and reproduction), the different functional units of the society are clearly segregated in multilevel species (Swedell and Plummer 2012). Socioecological theory provides a framework for an attempt to understand the functionality behind these structures, as the same factors that drive socialization in nonmodular species may also have explanatory power in a multilevel setting, e.g., predation avoidance (Matsuda *et al*. 2010; Yeager 1992; Whitehead *et al*. 2012), facilitation of foraging (Whitehead 2003), or conspecific threat (Grueter and van Schaik 2010). Each level has evolved in response to a different compilation of cost–benefit tradeoffs (Wittemyer *et al*. 2005), but any endeavor aimed at identifying the function of the different social tiers is complicated by the fact that we must distinguish among several targets of ultimate reasoning, i.e., 1) evolutionary pathways: multilevel systems can evolve as a result of splitting of large mixed-sex groups or an amalgamation of discrete family units, 2) the emergent factors keeping the system in place, and 3) the adaptive significance of fission–fusion (if prevalent) among constituent units. For example it is assumed that compression of large numbers of individuals into a localized resource in hamadryas baboons has created a harassment-prone environment leading to substructuring (evolution). Male bonds across units contribute to uphold the system (emergent factor), while the patchy nature of food resources and restricted availability of roosting sites leads to fissioning and fusioning (Grueter *et al*. 2012). There are examples of all three analytical approaches in this issue: Grueter *et al*. (2012) attempt to depict the most parsimonious evolutionary pathways to modularity in different primate lineages. A reconstruction of ancestral social states is always fraught with a degree of “paleo-poetry,” but the use of a combination of phylogenetic, morphological, peleoenvironmental, and socioecological evidence can significantly narrow down the options. Bowler *et al*. (2012) rely on proximity measures to identify the social organization of uakaris, whereas Zhang *et al*. (2012) rely on such measures to deduce affiliation patterns within OMUs of *Rhinopithecus*. Finally, Ren *et al*. (2012) describe fission–fusion dynamics within a society of *Rhinopithecus* and explain temporary group splitting with optimization of access to seasonal foods.

Given the tremendous variety of habitats in which multilevel sociality has evolved, pinning it down to a universal environmental driver is utopian. Environmental features can constrain the evolution of modularity (Grueter and van Schaik 2010), but modularity is also the outcome of individual behavioral strategies. The explanatory framework for multilevel sociality contains recurring themes that are of great importance for comparative primatologists and anthropologists, e.g., conspecific threat and infanticide (Grueter and van Schaik 2010; Henzi and Barrett 2003), band-level male–male cooperation (Hill *et al*. 2009; Jolly and Phillips-Conroy 1998), and a tolerance or alliance network among some groups caused by female exogamy (Chagnon 1992; Chapais 2008). Mutualistic benefits accrued through collective defense, i.e., experiencing a reduced probability of being challenged when with other units (Colmenares 2004; Rubenstein and Hack 2004), may have substantial explanatory power for the emergence of modularity. Evidence for cooperation in the defense of group integrity against satellite males is still circumstantial in multilevel societies (Dunbar and Dunbar 1975; Krzton 2011; Zhao and Li 2009), but this needs to be rigorously assessed through long-term field programs.

Studying the nuances of multilevel sociality in primates also has potentially important implications for hominin behavioral evolution. Chimpanzees may well be an apt referential model that helps us to characterize the social system of the last common ancestor (*cf*. McGrew 2010), but for the transformation of mixed sex groups to embedded pair bonds —the modal system in humans— we can learn a great deal from primates living in multilevel societies (Swedell and Plummer 2012). Male kin-bonding is another hallmark of human sociality that appears to have its analogue in hamadryas clans (Swedell and Plummer 2012). Rodseth (2012) devalues the importance of marital ties as the linchpin of human societies by raising awareness for the integral role that male social solidarity plays in human societies, with conjugal bonds sometimes being nothing but mere appendages to an all-male association. Layton *et al*. (2012) attempt to trace the appearance of band organization in modern human hunter-gatherers via inferences from brain evolution, ethnography, and the movement of stone tools beyond the source of their raw materials at various periods in prehistory.

Although hamadryas baboons, which are famous for their multilevel system, were among the first primate species to be studied in the wild (Kummer 1968, 1995), the dynamics of most multilevel societies are still poorly understood. There are many possible avenues for further research, both in the laboratory and the field. For some species, such as uakaris (Bowler *et al*. 2012), drills (*Mandrillus leucophaeus*: Astaras 2009; Astaras *et al*. 2008), doucs (*Pygathrix* spp.: Rawson 2009), and golden langurs (*Trachypithecus geei*: Mukherjee and Saha 1974), we have only limited field data on social organization and structure and much more empirical evidence for the existence of modularity is needed, as well as data on genetic relationships among individuals of all levels of a nested society. There is a clear need for more field studies on these neglected primate taxa. For colobines in particular, we need to gain a clearer understanding of the network structure and links across social levels within modular societies. The use of artificial approaches such as agent-based modeling may be another promising way to tackle the analysis of determinants of multilevel organizations (Zinner and Hammerschmidt 2008). The cooperative potential among coresiding males in multilevel societies may be latent, but might be activated by artificial stimuli (if ethically justifiable), as shown by actively coordinated group reactions to trapping (Jolly and Phillips-Conroy 1998).

Band formation in some multilevel species, e.g., colobines, has been argued to represent an adaptation to enhanced threat from conspecifics (Grueter and van Schaik 2010). Another possible explanation that has not yet received empirical scrutiny is that band formation may be a strategy to facilitate allocare in species living under precarious environmental conditions, resulting in mutualistic alleviation of female energetic stress. If this turns out to have explanatory power, then it may have implications for our understanding of the evolution of allocare and cooperative breeding in humans (Hrdy 2009). Almost untouched is the question of what predictions we have concerning individual recognition, group coordination, communication, cognitive skills, decision making, and disease transmission in multilevel organizations (Fischer and Zinner 2011; Sueur *et al*. 2011). Also remarkable and requiring explanation is that most or all primate species that live in large multilevel societies, e.g., geladas, snub-nosed monkeys, proboscis monkeys, douc langurs, exhibit striking facial and bodily coloration as well as other ornaments. These conspicuous signals may be adaptive in primates living in small cohesive groups where individual recognition is the primary means by which animals “know” one another. However, in large modular groups —particularly groups where membership is not always constant— individuals require alternative ways of “knowing” each another and assessing each other’s quality and status. It has been suggested (Bergman *et al*. 2009; Setchell and Kappeler 2003) that living in large groups selects for such conspicuous ornaments (presumably sexually selected signals of quality) because individual recognition is no longer an option.

It is obvious that our knowledge of these intriguing societies is still limited. Unfortunately, most species showing multilevel sociality are endangered, and pristine conditions allowing the complexities of multilevel systems to unfold unhampered are becoming rare. Bands of snub-nosed monkeys often occur in fragmented habitats with limited or absent connectivity with neighboring bands (Grueter *et al*. in press). Bands/populations of proboscis monkeys have become locally extinct as a result of habitat loss (Sha *et al*. 2008). Heavy modern whaling in the Pacific may have destroyed the integrity of many sperm whale social units (Whitehead *et al*. 2012). Poaching has altered the expression of the elephant social system (Foley 2002). We hope that this special issue will spark interest among primatologists, mammalogists and biocultural anthropologists to embark on studies pertaining to the structure and evolution of these multilayered societies and thereby not only contribute to a better understanding of the evolutionary drivers of human social evolution, but also raise awareness for the plight of this unique evolutionary heritage.
